# The Relationship Between Microbiota, Nutrition, and Gastrointestinal Tract Symptoms in Patients with Systemic Sclerosis: A Systematic Review of the Literature

**DOI:** 10.3390/ijms26167685

**Published:** 2025-08-08

**Authors:** Francesca Gori, Laura Tomaino, Carlo La Vecchia, Simona Servida, Luisella Vigna

**Affiliations:** 1 Department of Anesthesia, Critical Care and Emergency, Fondazione IRCCS Ca’ Granda-Ospedale Maggiore Policlinico, 20122 Milan, Italy; francesca.gori@policlinico.mi.it; 2 Emergency Medicine Residency Program, Marche Polytechnic University, 60121 Ancona, Italy; laura.tomaino@gmail.com; 3 Department of Clinical Sciences and Community Health (DISCCO), Università degli Studi di Milano, 20122 Milan, Italy; carlo.lavecchia@unimi.it; 4 Occupational Health Unit, Center of Obesity, Fondazione IRCCS Ca’ Granda-Ospedale Maggiore Policlinico, 20122 Milan, Italy

**Keywords:** systemic sclerosis, scleroderma, gastrointestinal involvement, nutrition, gut microbiota, diet, malnutrition

## Abstract

Gastrointestinal (GI) involvement is frequent in systemic sclerosis (SSc). Recent studies have highlighted a possible association between GI symptoms, gut microbiota (GM), and nutrition. In this systematic review, the evidence supporting these relationships was evaluated. Articles selected from the MEDLINE database and published between 2010 and 2025 were included. Keywords used in the sources included “systemic sclerosis”, “gut microbiota”, “malnutrition”, and “gastrointestinal symptoms”. Forty-nine articles were selected, for a total of 6270 SSc patients. The evidence suggests a complex relationship between SSc, GI symptoms, and GM dysbiosis. Both are manifestations of the disease, and in turn they influence its severity. Digestive tract fibrosis and dysbiosis alter nutrient absorption, which can lead to malnutrition. However, data considering these complex relationships between the GI tract, microbiome, and nutritional status are few and very heterogeneous. Further studies are needed to investigate these complex interwinings and identify nutritional interventions able to reduce GI dysfunction and simultaneously counteract malnutrition.

## 1. Introduction

Systemic sclerosis (SSc), also known as scleroderma, is a connective tissue disorder characterized by obliterative microvasculopathy, early activation of leukocytes, atrophy of smooth muscles, and diffuse fibrosis of the skin and internal organs. SSc implies multisystem involvement, heterogeneous clinical manifestations, and a progressive course, with substantial disability and mortality [1]. Like other autoimmune diseases, SSc is likely the result of a combination both of host genetic and environmental factors. The skin, heart, lungs, kidneys, and digestive tract are the sites most frequently affected by the disease [2,3,4]. Gastrointestinal (GI) tract clinical manifestations affect about 90% of the cases and include esophageal reflux, dysphagia, postprandial bloating, abdominal distension and pain, diarrhea, and malabsorption, which can lead to severe malnutrition, constipation, and fecal incontinence [5]. The heterogeneity of GI symptoms complicates the clinical approach, and the quality of life and life expectancy of the patient are greatly affected. A recent review attempted to summarize the clinical, serological, and genetic risk factors associated with GI tract complications [2], which are currently evaluated by screening for small bowel hypomotility, nutritional status, small intestinal bacterial overgrowth (SIBO), and anorectal dysfunction. Dysfunctions of the digestive tract, particularly those in the small intestine (hypomotility, atrophy, and fibrosis), lead to stasis of the small bowel, and thus to bacterial colonization that can result in SIBO. Growing evidence highlights differences in the gut microbiota (GM) between healthy subjects and SSc patients, underscoring that the GM may represent an important mediator in the pathogenesis of this autoimmune disease. At the same time, some studies suggest that differences in GM composition between SSc patients and healthy controls (HCs) may be related to the severity of GI symptoms of the disease [6,7]. GI tract dysfunction and altered bacterial colonization are closely linked and both considered clinical manifestations of SSc. Both, however, can lead to a worsening of the disease, on the one hand inducing dysfunction and fibrosis of the visceral organs, vascular damage, and inflammation [2,8] and, on the other hand, altering the microbiome, intensifying the inflammatory processes, and influencing metabolism [9].

The interventions currently applied to reduce GI symptoms do not appear to be adequate. The range of interventions can include a targeted diet in patients with diarrhea, the use of antibiotics against SIBO, bile salts, and pancreatic enzymes against malabsorption, medical nutritional support in cases of severe malnutrition, immunomodulatory therapies, and minimally invasive surgery. This systematic review focused on typical GI symptoms present in patients with SSc, with the aim of investigating the relationship between these, the GM, the nutritional status, and the nutritional interventions currently available to counteract the development of GI dysfunction. Finally, we analyzed whether GI symptoms, GM alteration, and malnutrition could be predictors of mortality in these patients.

## 2. Materials and Methods

### 2.1. Information Sources and Search Strategy

**Figure 1:**
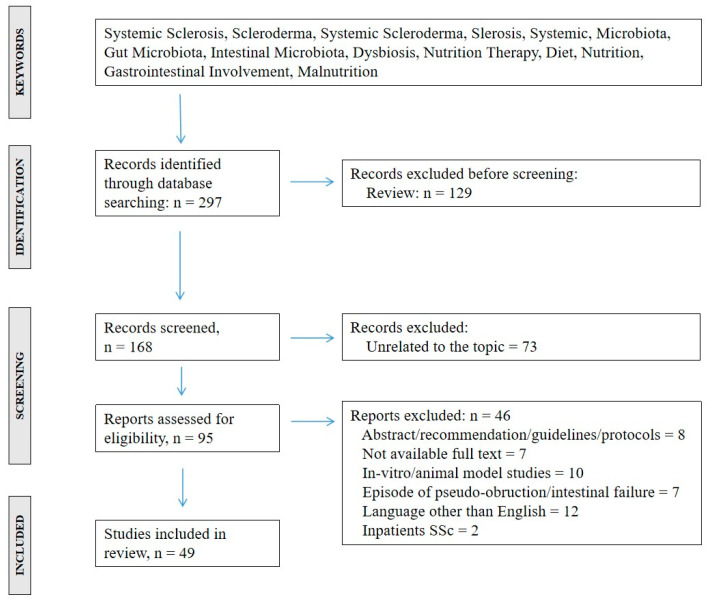
Search flow diagram.

A systematic review of the literature was conducted according to the PRISMA (Preferred Reporting Items for Systematic reviews and Meta-Analyses) recommendations [10]. Published studies on the topic were retrieved after a search in MEDLINE, from January 2010 to May 2025. Only English-language articles were included. To identify relevant records, we defined a research string including “systemic scleroderma”, “microbiota”, “gut microbiota”, “intestinal microbiota”, “dysbiosis”, “nutrition therapy”, “diet”, “nutrition”, and “malnutrition” (Figure 1). In the search, we included both potential causative and outcome events. A critical evaluation was subsequently performed by two authors (L.T. and F.G.). The systematic review protocol is available through a public register (PROSPERO, n 2025 CRD420251090401).

### 2.2. Eligibility Criteria

Studies meeting the following criteria were included: (1) studies (i.e., cross-sectional, case report, case–control) addressing GI symptoms in patients with a definite diagnosis of SSc; (2) studies published in English; and (3) studies involving non-hospitalized adult patients. The exclusion criteria were (1) abstracts, guidelines, protocols, recommendations, reviews, or editorials; (2) in vitro and preclinical studies; and (3) studies involving inpatients or severe GI disease with intestinal pseudo-obstruction or intestinal failure.

### 2.3. Selection Process

After performing the systematic research, and removing duplicate records and review articles, two reviewers (L.T. and F.G.) independently screened the remaining records, checking titles and abstracts. At the end of the screening process, the reviewers shared their evaluations, and, in case of discrepancy, the entire article was appraised and re-evaluated for selection. No automation tool was used in this phase.

### 2.4. Data Extraction, Date Items, and Result Synthesis

After the application of the inclusion and exclusion criteria and after reviewers’ consensus, a report of the included records was developed. Selected papers were evaluated and analyzed, and data regarding study design, sample, intervention, results, and other relevant information were extracted and reported in tables. No automation tool was used in this process.

### 2.5. Quality Assessment

No tool was used to assess the quality of the identified studies. It was evaluated by two researchers independently, and the discrepancies were discussed together and resolved. The PRISMA checklist for systematic reviews is presented in Online Supplementary Table S1.

## 3. Results

### 3.1. Study Selection

A total of 297 records were initially identified. After a first manual evaluation, 129 records were excluded and 93 full-text articles were further evaluated for eligibility. Finally, 49 studies fulfilled the inclusion criteria, as shown in Figure 1.

### 3.2. Study Characteristics

Table 1, Table 2, Table 3, Table 4 and Table 5 summarize the included studies [8,11,12,13,14,15,16,17,18,19,20,21,22,23,24,25,26,27,28,29,30,31,32,33,34,35,36,37,38,39,40,41,42,43,44,45,46,47,48,49,50,51,52,53,54,55,56,57,58], which were classified as follows: studies [11,12,13,14,15,16,17,18] on the relationship between the GM and GI symptoms (Table 1); studies [19,20,21,22,23] on the effects of the probiotic treatments and fecal microbiota transplantation (FMT) (Table 2) on GI symptoms; studies [8,24,25,26,27,28,29,30,31,32,33,34,35,36,37,38,39,40,41,42] focused on the relationship between malnutrition and GI symptoms (Table 3); studies [17,43,44,45,46,47,48,49,50,51,52] regarding the effects of nutritional intervention in SSc patients (Table 4); and studies [38,53,54,55,56,57,58] on predictor factors of mortality (Table 5). Among selected studies, 4 were randomized controlled trials (RCTs), 18 were prospective studies, 18 were retrospective studies, 1 was a case report, and 9 were cross-sectional studies. A total of 6270 patients with SSc and 1019 HCs were included. Most patients diagnosed with SSc included in the studies were women (at least 55.5%, though gender distinction was not always present), with an average age of 53.6 ± 5.7 years and an average duration of disease of 11.9 ± 8.7 years.

### 3.3. Relationship Between Gut Microbiota and Gastrointestinal Involvement

Eight studies [11,12,13,14,15,16,17,18] assessed the GM differences between SSc patients and HCs (Table 1). A total of 458 patients and 269 HCs were analyzed and compared. All studies highlighted that patients with SSc had a state of dysbiosis (up to 75.5% of cases), characterized by an increase in *Lactobacillus* [11,14], *Streptococcus* [14], *Parabacterioides* [13], *Butyricimonas* [13], proinflammatory noxious genera (especially *Desulfovibrio*) [13], *Bifidobacterium* [11], and pathobionts (*Fusobacterium* [11], *g-Proteobacteria* [11], *Ruminococcus* [11], *Akkermansia* [11], *Klebsiella* [17], and *Enterococcus* [17]). At the same time, the GM of SSc patients was also characterized by a lower presence of *Sutterella* [14], butyrate-producing bacteria (*Turicibacter* and unidentified members of the Lachnospiraceae family) [11], and commensal bacteria (*Faecalibacterium* and *Clostridium*) [11] compared with HCs. An enrichment of *Fusobacterium*, *Ruminococcus,* and *Akkermansia* genera has also been identified in SSc patients compared to unaffected controls [12,13]. An Italian study [18] compared the GM composition of patients with a very early diagnosis of systemic sclerosis (VEDOSS) with patients with definite SSc and HCs. When comparing HCs with VEDOSS patients, there was an increase in the Bacteroidales and Oscillospirales orders, the Oscillospiraceae family, and the *Anaerostipes* genus and a decrease in the Bacilli and Alphaproteobacteria classes, the Lactobacillales order, and the *Blautia*, *Romboutsia*, *Streptococcus*, and *Turicibacter* genera. Instead, patients with definite SSc showed an elevated number of the Acidaminococcaceae and Sutterellaceae families and a decrease in the Peptostreptococcaceae family and the *Anaerostipes*, *Blautia*, *Romboutsia*, and *Turicibacter* genera compared to HCs.

Although the food intake and lifestyle affect the GM and its composition, the GM found in SSc patients did not depend on nutritional intake, with the exception of the unidentified members of the Erysipelotrichaceae family, which were related to short-term dietary modifications [13]. GM produces metabolites that can influence health and host homeostasis. Therefore, depending on the bacteria present, the metabolites circulating in the serum will vary, and alterations in this relationship have been linked to specific diseases. In Italian SSc patients, Bellocchi et al. [13] found several associations between *Desulfovibrio* and the production of benzene derivatives, glycerophospholipids, and derivatives of amino acid metabolism. Additionally, patients with SSc and VEDOSS, compared to HCs, had lower concentrations of butyrate and higher concentrations of acetate [18]. Metabolite production also appears to differ according to the degree of the disease or its duration. Bellando-Randone and colleagues [18] found alterations in acetate and valerate concentrations in relation to the duration of the disease. Specifically, in VEDOSS, acetate and valerate values were higher and lower, respectively, than in patients with definite SSc. However, many of these associations require further investigation to provide convincing evidence.

Four articles [11,12,15,16,17,18], in addition to defining the composition of the GM in SSc, evaluated the link between dysbiosis and SSc-GI symptoms in SSc. Besides having a different GM compared to HCs, patients with GI symptoms had an increase in *Lactobacilli*, *Eubacterium*, *Acinetobacter*, *Klebsiella*, and *Enterococcus* and a decrease in *Roseburia*, *Clostridium*, and *Rinococcus*. In cases of SSc, but without GI symptoms, the GM composition was more similar to that of HCs, except for a high presence in *S. salivarius* compared to those with GI disorders [12]. In Volkmann et al.’s [11] study, when comparing SSc patients with and without GI involvement, patients with moderate-to-severe GI symptoms had a reduction in *Bacteroides fragilis* and an increase in *Fusobacterium*. In a subsequent longitudinal study of SSc patients, reductions in the commensal species *Bacteroides fragilis* were associated with worsening GI tract symptoms over time, even after adjusting for the disease duration and disease subtype [15].

New research has confirmed that this relationship is not unidirectional and that the GM is not only influenced by disease progression but can also in turn influence the intestinal motor complexes. In the study by Andreasson et al. [16], patients with recent-onset SSc (median disease duration of two years) had an increase in pathobiontic genera (*Desulfovibrio* and *Ruminococcus*) and a reduction in a commensal genus (*Faecalibacterium*) compared to age- and sex-matched controls. Very few patients had clinical signs of SSc-related lower-GI-tract involvement. In that study [16], differences in microbial composition emerged between SSc patients with interstitial lung disease (ILD) and those without. Furthermore, differences in microbial composition have been reported between patients with and without SIBO. In multivariate analysis, ILD was significantly associated with microbial composition. It is unclear whether these GM alterations contribute to the pathogenesis of ILD in SSc; however, these early findings provide evidence of a link between distinct microbiome profiles and specific clinical manifestations of SSc.

Dysbiosis could therefore play an important role in the progression and severity of the disease. Since the composition of the GM varies in relation to the degree of the disease itself [14], the re-establishment of a state of eubiosis may bring benefits [14] and better management of the pathology. Since butyrate-producing bacteria are deficient from the early stages of SSc, a possible therapeutic approach to alleviate GI symptoms could be the supplementation of butyrate from the beginning of the disease diagnosis [17], whereas a non-low fermentable oligoaccharide, disaccharide, monosaccharide, and polyols diet was not associated with alterations in GM composition or a reduction in GI symptoms [17].

**Table 1:** Studies on the relationship between gut microbiota and gastrointestinal symptoms.

Ref	Sample Size	Age (yrs)	Comparison Group	GM Composition	GM and GI Symptoms’ Relation
[11]	17 SSc	52.1 [46.6–63.0]	SSc vs. HCs	↑ *Fusobacterium,* *γ-Proteobacteria* (potential pathobiont) ↑ *Bifidobacterium, Lactobacillus* *↓ Faecalibacterium, Clostridium* (commensal bacteria)	N/A
	17 HCs	55.0 [51.0–62.0]	SSc/GI**^+^** vs. SSc/GI**^−^**	N/A	↑*Fusobacterium* ↓*Bacteroides fragilis*
[12]	9 SSc/GI^+^	55.3 [36–79]	SSc/GI**^−^** vs. HCs	↑ *Streptococcus salivarius*	N/A
	9 SSc/GI^−^	57.4 [34–78]	SSc/GI**^+^** vs. HCs	N/A	↑ *Lactobacillus, Eubacterium, Acinetobacter* ↓ *Roseburia, Clostridium, Ruminococcus*
	9 HCs	54.8 [26–78]	SSc/GI**^−^** vs. SSc/GI**^+^**	N/A	↑ *Streptococcus salivarius*
[13]	59 SSc	56.5 ± 12.7	SSc vs. HCs	↓ protective butyrate-production bacteria ↑ proinflammatory noxius genera (*Desulfovibrio*)	N/A
	29 HCs	49.3 ± 12.9	SSc/GI**^+^** vs. HCs	N7A	10 different genera between HCs, SSc/GI**^+^**, and SSc/GI
[14]	63 SSc	52.5 ± 14.2	SSc vs. HCs	↑*Firmicutes philum, Streptococcus, Lactobacillus, Blautia, Ruminococcus, Phascolarctobacterium* genera ↓*Sutterella, Bacteroides, Odoribacter, Roseburia* genera	N/A
	17 HCs	50.8 ± 14.3			
[15]	19 SSc	51.3 [48.7–59.4]		↑ *Bacteroides*, *Prevotella*, genera from the Clostridiales order	*Bacteroides* → Total GI symptoms *Prevotella* → Diarrhea Genera from Clostridiales order →Distension/ bloating
[16]	106 SSc 85 HCs	55.3 ± 15.9	SSc vs. HCs	↓ Commensal genera (e.g., *Faecalibacterium*) ↑ Pathobiont genera (e.g., *Desulfovibrio*)	SIBO as indicator of dysbiosis in several sites of GI tract
[17]	66 SSc	55.4 ± 11.8	SSc/GI^+^ vs. SSc/GI^−^	N/A	↑ *Klebsiella, Enterococcus*
			Non-low FODMAP vs. low FODMAP	↑ *Enterococcus*	Similar microbial composition and GI symptoms
[18]	26 SSc	64.8 ± 11.9	SSc vs. HCs	↑ Acidaminococcaceae and Sutterellaceae families ↓Peptostreptococcaceae family, *Anaerostipes, Blautia, Romboutsia,* and *Turicibacter* genera	N/A
	18 VEDOSS	51.7 ± 16.1	VEDOSS vs. HCs	↓ Bacilli class, *Blautia, Romboutsia, Streptococcus,* *Turicibacter*	N/A
	20 HCs	50.8 ± 14.3	VEDOSS vs. SSc	N/A	N/A

↑: increase; ↓: reduction; LOW-FODMAP: fermentable oligosaccharides, disaccharides, monosaccharides, and polyols (FODMAP) diet; GI: gastrointestinal symptoms; GM: gut microbiota; HCs: healthy controls; SSc: systemic sclerosis; VEDOSS: very early diagnosis of SSc.

Of the studies examined in this review, three papers [19,20,21] assessed the effectiveness of probiotic treatment to reduce GI symptoms (Table 2). In two of these [20,21], the diagnosis of SSc patients was performed according to the 2013 American College of Rheumatology/European League Against Rheumatism (ACR/EULAR) criteria, while all three studies used the University of California Los Angeles Scleroderma Clinical Trials Consortium Gastrointestinal Tract 2.0 (UCLA GIT 2.0) scale to define GI involvement and its impact on quality of life. The studies had different administration times: 60 [20,21] and 120 days [19,20]. When comparing these studies, the results were controversial. In Low et al.’s study [20], an improvement in GI symptoms was noted after 120 days of treatment, but only in relation to reflux, while in Frech’s et al.’s study [19], reflux was reduced, but the main effect was recorded on bloating/swelling. In Marighela et al.’s study [21], an improvement in the UCLA GIT 2.0 total score and in the Health Assessment Questionnaire-Disability Index score was reported, together with an immunomodulatory effect, especially a stimulation of Th1, Th2, and Th17 lymphocytes and regulation of T cells, but there was no beneficial effect on GI symptoms. Another possible treatment to relieve GI symptoms is FMT. The efficacy and safety of FMT using standardized anaerobic cultivated human GM was evaluated in two studies conducted by the same research group for the treatment of symptoms of the lower GI tract [22,23]. They were a double-blind, placebo-controlled, randomized pilot trial [22] and a phase-2, randomized, double-blind, placebo-controlled trial [23]. However, the results regarding the reduction in GI symptoms were mixed. In the pilot study [22], an effective reduction in GI symptoms was reported, probably due to a modification of the GM composition, accompanied by mild and transient side effects (gastroduodenoscopy complications). In contrast, in the phase-2 study [23], after 12 weeks of intervention, no change in the most severe symptoms of the lower GI tract was recorded. This study also observed mild and short-lived adverse events affecting the GI tract. In only one case, during gastroscopy, a duodenal perforation occurred.

**Table 2:** Studies on the effects of the probiotic treatment and fecal microbiota transplantation on gastrointestinal symptoms.

Ref	Study Design	Sample Size	Age (yrs)	SSc Severity	Intervention Group n (Females)	Comparison Group n (Females)	Treatment Duration	Effects on GI Symptoms
[19]	Perspective study. Participants were supplemented with either Align (*Bifidobacterium infantis*; 10^9 CFU per capsule) or Culturelle (*Lactobacillus GG*; 10^9 CFU per capsule) once a day	10 SSc	51.7	UCLA STCT GIT 2.0 total score 1.25–3.00	10 (9)	-	2 months supplementation	Improvement in total GIT 2.0 score, reflux, bloating/distention, and emotional scales
[20]	RCT placebo controlled. Participants were randomized (1:1) to receive 60 days of high dose multi-strain probiotics (Vivomixx 1800 billion units/day) or identical placebo, followed by an additional 60 days of probiotics in both groups	40 SSc		UCLA STCT GIT 2.0 total score > 0.10	19 (19)	21 (16)	60 + 60 days	No change in UCLA STCT GIT 2.0 after 60 or 120 days of treatment Significant improvement in GI-reflux after 120 days
		21 Placebo-Probiotics	50.7 ± 8.0					
		19 Probiotics-Probiotics	51.4 ± 13.7					
[21]	RCT placebo controlled. Participants were randomly assigned to receive a daily dose of probiotics (*Lactobacillus paracasei, Lactobacillus rhamnosus, Lactobacillus acidophillus,* and *Bifidobacterium lactis*, 10^9 CFU per capsule) or placebo for 8 weeks	73 SSc		UCLA STCT GIT 2.0 total score > 0.50	37 (34)	36 (34)	8 weeks	No difference in the UCLA STCT GIT 2.0 score after 8 weeks Probiotic group: ↓ Th17 No difference in the Th1, Th2 and regulatory T cells No difference in HAQ-DI score
		37 Probiotic group	46.7 ± 13.1					
		36 Placebo	47.1 ± 11.9					
[22]	A double-blind, placebo-controlled pilot study	9 SSc	62.0 ± 5.7	UCLA STCT GIT 2.0 total score 0.72 ± 0.5	5 FMT	4 Placebo	16 week after FMT	FMT with ACHIM reduces lower GI symptoms, altering the GM
[23]	A phase 2 randomised, double-blind, placebo-controlled study	67 SSc	58.9 ± 11.5	UCLA STCT GIT 2.0 total score 0.9 ± 0.5	33 (33) ACHIM	34 (29) Placebo	12 week after FMT	No change in lower GI symptoms after FMT

ACHIM: anaerobic cultivated human intestinal microbiome; FMT: fecal microbiota transplant; RCT: randomized controlled trial; SSc: systemic sclerosis patients; UCLA STCT GIT 2.0: University of California Los Angeles Scleroderma Clinical Trials Consortium Gastrointestinal Tract 2.0; GI: gastrointestinal; CFU: colony forming unit; HAQ-DI: Health Assessment Questionnaire—Disability Index.

### 3.4. Relationship Between Malnutrition and Gastrointestinal Involvement

The evaluation of the nutritional status is fundamental in patients with SSc, since malnutrition leads to anthropometric and blood alterations (including a decrease in prealbumin), which indicate the involvement of various organs, mainly the skin and the GI tract [59]. Table 3 shows the studies focusing on malnutrition in SSc patients. Of the 49 manuscripts considered in this review, 20 articles [8,24,25,26,27,28,29,30,31,32,33,34,35,36,37,38,39,40,41,42] evaluate the prevalence of malnutrition [8,24,26,27,28,29,30,31,32,33,34,35,36,37,38,39,40,41,42] and its association with activity [24,36] and the severity of disease [27,35,36], bone alterations [31,34], GI symptoms [8,27,28,33,38,39,40,41,42], serum prealbumin [24,30] and albumin [41] levels, depression [34], sarcopenia [27,29], low muscularity [27,37], microvasculopathy [33], cardiac [28] or lung disease [27,32,34], hospitalization [38], quality of life [25,26,40], and mortality [38]. Although some authors believe that its prevalence was lower than that of other chronic and autoimmune diseases with GI involvement [36], malnutrition in SSc patients was present in variable percentages depending on the diagnostic method used. The most common criteria used were the Malnutrition Universal Screening Tool (MUST) [8,26,28,32,34,35] and European Society of Clinical Nutrition and Metabolism (ESPEN) [27,29,31,33,36,37], but the Global Leadership Initiative on Malnutrition (GLIM) [33,36,37,38,39,41], Short Nutritional Assessment Questionnaire (SNAQ) [33], and Subjective Global Assessment, 1–7 points (7-SGA) [8,33] have also been used, even comparing them with each other [36,37], with the result that GLIM detected a greater prevalence of malnutrition than with ESPEN criteria (16.6% vs. 8.8%) [36]. This was also confirmed by two other studies in which ESPEN [27,33], SNAQ, and 7-SGA [33] underestimated the malnutrition status when compared with GLIM. In Wojteczek et al.’s study [33], the percentages of patients with an altered nutritional status were 16.1%, 17.9%, 23.2%, and 62.5% according to SNAQ, ESPEN, 7-SGA, and GLIM criteria, respectively.

With the exception of some studies [24,25,30,34,36,37], the association between malnutrition and GI symptoms seems to be confirmed: the more severe the degree of malnutrition, the more severe the GI symptoms. Moreover, some selected micronutrient deficiencies, such as calcium and 25OH-vitamin D, which described the bone status [31], but not zinc, selenium, and folic acid [30], were associated with GI involvement in SSc patients.

**Table 3:** Studies with malnutrition topic.

Ref	Total Sample and Subgroups	Age (yrs)	Criteria, and Total Score of GI Involvement	n (%) of GI Involvement	Malnutrition Criteria	Malnutrition Associations
[8]	24 SSc	54 ± 13	UCLA SCTC GIT 2.0	17 (71)	MUST SGA	Malnutrition risk prevalence: 37.5% (MUST) Malnutrition prevalence: 50% (SGA) Malnutrition status was associated with total UCLA SCTC GIT 2.0 score, reflux, distention/bloating, soilage, diarrhea, social function, and emotional well-being
[24]	160 SSc	61.7 [53.9–68.0]	Medsger's severity scale	82 (51.2)	BMI	Malnutrition prevalence: 15% Malnutrition was associated with disease activity, low serum prealbumin, but not with GI involvement
	24 Malnourished patients	61.7 [54.0–67.9]		16 (66.7)		
	136 No- malnourished patients	61.1 [47.2–68.5]		66 (48.5)		
[25]	52 SSc 51 HCs	N/A	N/A	N/A	BMI	SSc patients have a lower BMI BMI was associated to quality of life, but not to GI symptoms. GI symptoms was associated with life quality No associations between low BMI and upper GI symptoms (heartburn, nausea, vomiting, dysphagia, and epigastric pain)
[26]	129 SSc	59.1 ± 13.8	N/A	N/A	MUST	Malnutrition risk prevalence: 10.9%. Malnutrition was associated with the quality of life
[27]	141 SSc	63 ± 13	UCLA SCTC GIT 2.0	16 (11.3)	ESPEN	Malnutrition prevalence: 9.2% Sarcopenia prevalence: 20.7% Malnutrition is associated with GI symptoms, low muscularity, reduced FVC, and DLCO Malnutrition and sarcopenia affect disease severity, especially for the lung
[28]	120 SSc	64 ± 15	N/A	111 (93)	MUST PNDS	Malnutrition prevalence: 59.2% Malnutrition risk: 58% Malnutrition was associated with cardiac involvement, number of GI organs involved, gastroesophageal reflux disease, chronic intestinal pseudo obstruction
[29]	62 SSc	62 [32–78]	N/A	26 (42) esophageal involvement	ESPEN	Malnutrition prevalence: 19% Sarcopenia prevalence: 42% (RSMI) and 55% (HGS) Sarcopenia was associated with esophageal involvement
[30]	176 Established SSc	58 [49–67]	UCLA SCTC GIT 2.0	231 (92.4)	EUSTAR	Malnutrition prevalence: 48.3% Micronutrients deficiences were very common, especially in folic acid, selenium, prealbumin, and zinc Micronutrient deficiencies was associated with low BMI, but not with GI involvement, except for prealbumin
	74 early SSc	52 [38–61]				
[31]	36 postmenopausal female patients with SSc	N/A	N/A	12 (33)	ESPEN	Malnutrition prevalence: 36.1% ↓weight, BMI, hemoglobin, albumin, parathyroid hormone, 25-hydroxyvitamin D, lumbar spine T-score, bone mineral density values, and trabecular bone score values Bone status correlated with serum biomarkers of malnutrition, and GI symptoms
	13 Malnourished patients	54 [43–75]				
	23 Non-malnourished patients	66 [43–85]				
[32]	134 SSc	50.2 ± 12.3	UCLA SCTC GIT 2.0 (0.24 ± 0.27)	20 (15)	MUST	Malnutrition risk prevalence: 15% Malnutrition was associated with limited pulmonary function, lung involvement, pulmonary hypertension, capillary rarefaction Capillary rarefaction was independently associated with medium to high malnutrition risk Capillary rarefaction and severe skin involvement were determining factors for malnutrition and GI symptoms
[33]	56 SSc	54.1 ± 11.9	N/A	43 (76.8)	ESPEN 7-SGA SNAQ GLIM	Malnutrition prevalence: 17.9% (ESPEN 2015), 23.2% (7-SGA), 16.1% (SNAQ), and 62.5% (GLIM) Malnutrition was associated with GI symptoms
[34]	98 SSc	52.7 ± 11.3	N/A	14 (14.6)	MUST	Malnutrition prevalence: 100% (including both in- and outpatients) Malnourished patients had high mRSS Malnutrition risk was associated with interstitial lung disease and bowel involvement, and depressive symptoms, but not with GI symptoms
[35]	168 SSc	61 [25–81]	UCLA SCTC GIT 2.0 (0.9 ± 0.9)	N/A	MUST	Malnutrition prevalence: 16% BMI was associated with small intestinal involvement and disease severity
[36]	102 SSc	55 ± 14	N/A	N/A	GLIM ESPEN	Malnutrition prevalence: 8.8% (ESPEN), 16.6% (GLIM) According to GLIM, disease activity index and disease severity scale were associated with malnutrition, but not for ESPEN Malnutrition was not associated with GI symptoms
[37]	60 SSc	53 [43–63]	UCLA SCTC GIT 2.0 (0.53 [0.19–0.89])	9 (13)	ESPEN GLIM	Malnutrition prevalence: 11.6% (ESPEN), 23.2% (GLIM) FFMI, but not malnutrition, was associated with GI symptoms (distension/bloating)
[38]	101 SSc	55 [47–66]	N/A	10 (9.9)	GLIM	Malnutrition prevalence: 21.8% Malnutrition according GLIM criteria was associated with GI symptoms, hospitalization, and survival
[39]	100 SSc	62 [53–70]	UCLA SCTC GIT 2.0 (0.18 [0.04–0.41])	20 (20)	GLIM	Malnutrition prevalence: 29% Malnourished patients had increased GI symptoms. GI symptoms was associated with depression score (cognitive impairment prevalence: 50% according Montreal Cognitive Assessment). Malnutrition was associated with age, dysphagia, and mRSS
[40]	75 SSc	59.6 ± 10.6	UCLA SCTC GIT 2.0 (0.5 [0.2–1.1])	44 (58)	MNA- SF	Malnutrition prevalence: 30.7% Malnutrition was associated with severe GI symptoms, poor quality of life, and skin involvement
[41]	1903 SSc	47.3 [36.4–57.0]	UCLA SCTC GIT 2.0 (0.5 [0.2–1.0])	1389 (73)	GLIM	Malnutrition prevalence: 34% Malnutrition was associated with GI involvement, multimorbidity, cardiopulmonary disease, inflammation, hypoalbuminaemia, and anaemia
[42]	82 SSc	49.4 ± 13.0	UCLA SCTC GIT 2.0 (0.50 [0.04–1.49])	51 (62.2)	CONUT PNI	CONUT score: 1.45 ± 1.35 PNI: 43.59 ± 5.01 Malnutrition, as measured by CONUT and PNI, was associated with GI involvement

↓: reduction; BMI: body mass index; CONUT: Control of Nutritional Status score; DAI: disease activity index; DLCO: diffusing capacity for carbon monoxide; DSS: disease severity scale; ESPEN: European Society of Clinical Nutrition and Metabolism; FFMI: Fat-Free Mass Index; FVC: reduced forced vital capacity; GI: gastrointestinal symptoms; GLIM: Global Leadership Initiative on Malnutrition criteria; GSS: global symptomatic score; HGS: hand grip strength; HCs: healthy controls; MNA-SF: Mini Nutritional Assessment Short Form; N/A: not applicable; PNDS: Protocole National de Diagnostic et de Soins; mRSS: modified Rodnan skin score; MUST: Malnutrition Universal Screening Tool; PNI: Prognostic Nutrition Index; RSMI: Relative Skeletal Mass Index; SGA: Subjective Global Assessment; SSc: systemic sclerosis patients; UCLA STCT GIT 2.0: University of California Los Angeles Scleroderma Clinical Trials Consortium Gastrointestinal Tract 2.0.

### 3.5. Effects of the Nutritional Intervention on Gastrointestinal Involvement

GI symptoms are common in SSc patients (80% [60]) and although they are rarely the direct cause of death, they have an important impact on quality of life, leading to numerous comorbidities, which influence survival. There is no specific nutritional treatment to alleviate GI symptoms: although it has been highlighted that neither malnutrition [24] nor GI involvement [25] influences food intake, only a few clinical studies have evaluated a nutritional intervention. The nutritional interventions applied, some of which also led to an effective improvement in GI symptoms, were a targeted diet [43], provision of dietary counseling [44], with [45] or without [46] oral nutrition support (ONS), and artificial nutritional support [47,48,49,50,51,52], i.e., enteral nutrition (EN) and parenteral nutrition (PN) (Table 4). In Marie et al.’s study [43], participants with fructose intolerance, defined by a rise of H_2_ and/or CH_4_ levels above 20 p.p.m. on the fructose breath test, were prescribed a low-fermentable oligosaccharide, disaccharide, monosaccharide, and polyol diet for 1 month. After a good diet adherence, a reduction in GI symptoms, especially nausea, vomiting, abdominal pain, bloating, diarrhea, and abdominal tenderness, was recorded. However, Nguyen et al. [17], with the same type of diet applied for a period of 10 days, did not obtain any reduction in symptoms. The importance of personalized nutritional counseling in an interdisciplinary context has recently been reevaluated [44]. However, only two studies [45,46] investigated dietetic counseling in SSc patients, with [45] and without [46] ONS. Both studies concluded that the application of dietary counseling, even associated with ONS, could improve the nutritional status. Malnutrition was evaluated with MUST [45] or using the abridged Patient-Generated SGA [46]. Both after 6 weeks [45] and 1 year [46] of the nutritional intervention, an increase in body mass index (BMI), weight, and energy intake was recorded in both studies. Significant decreases in nutrition symptom scores and improvements in sarcopenia (expressed as lean mass/height^2^) were observed in Doerfler et al.’s study [45]. The levels of serum micronutrients increased in Ortiz-Santamaria et al.’s study [46], but not in Doerfler et al.’s study [45], whereas no improvement in GI symptoms was recorded. Only one case report has reported on EN in SSc patients [48], while PN was applied in six articles [47,48,49,50,51,52]. In four of these, malnutrition was assessed before PN treatment using the BMI [47,49], SGA [52], or Nutrition Risk Screening system [52]. The weight change was the main parameter that improved. Patients were continuously treated for 12 months [49,51,52] or at intervals of 3, 6, and 12 months and 7 years [48]. Grabowski and Grant [48] assessed the variation in mid-arm circumference and arm muscle area, without a clear finding, whereas five papers [47,48,49,51,52] reported complications after PN application, including catheter-related infection [47,49,51,52], calcification of the line [49,51], and reduction in autonomy, due to the request for help in the daily management of PN [47,49,51,52]. Overall, quality of life, measured by the Short Form Survey questionnaire [52] or Karnofsky Performance Status Score [50], improved for all patients involved. No deaths were attributed to the use of PN, but there were deaths attributed to complications related to the progression of the disease.

**Table 4:** Effects of the nutritional intervention on gastrointestinal involvement.

Ref	Study Sample	Age (yrs)	Study Type (Years)	Nutritional Intervention Type	Nutritional Intervention Duration	Main Results
[17]	66 SSc	55.4 ± 11.8	Prospective study (N/A)	Low-FODMAP	10 days	Similar microbial composition and GI symptoms
[43]	80 SSc		Prospective observational study (2011–2014)	Low fructose diet	1 month	Fructose malabsorption: 40% Correlation between fructose malabsorption and GSS score and absence of delayed gastric emptying Low-FODMAP: ↓ nausea, vomiting, abdominal pain, bloating, diarrhea, abdominal tenderness, and GSS score
	32 Fructose malabsorption	54 [27–79]				
	48 no-Fructose malaborption	53 [22–79]				
[44]	42 SSc	52 ± 12	Prospective observational study (N/A)	Nutritional counseling	N/A	↑> sodium intake Close association between dietary factors and body composition and GI symptoms. Malnutrition and weight loss were associated with pulmonary hypertension, heart failure, serum albumin, and skin fibrosis, but not advanced age
[45]	18 SSc	51 ± 11	Prospective study (N/A)	ON support	6 week + usual medical therapy	↓Nutrition symptom scores (12.8 vs. 7.6), and sarcopenia (54% vs. 39%) ↑ Appendicular lean height (5.6 ± 0.8 vs. 5.8 ± 0.8 kg/m^2^) No changes in caloric intake (1400 vs. 1577 kcal/d) and macronutrient distribution (% fat, protein, carbohydrate)
[46]	10+ SSc	63 ± 12	Prospective study (N/A)	Counselling + ON support	12 months	Dietary intervention maintained body weight and food intake No change in nutritional biochemical parameters, psychopathology and quality of life
[47]	15 SSc	N/A	Retrospective study (1979–1987)	PN support	2–90 months	↑ Quality of life Complications: catheter-related septicaemia (13%), superior vena cava obstruction (13%). 46% dead, none directly from GI disease or from the PN
[48]	5 SSc	-	Cases Reports	PN (4 cases) and EN (1 case) support	PN: 12–86 months EN: 16 months	↑ nutritional status, BMI, quality of life 60% dead from their disease
[49]	8 SSc	51 [42–56]	Retrospective comparative study (1993–2006)	PN support	40 [0.8–192] months	Complication: catheter-related infection (25%), line infection (25%), loss of autonomy (75%)
[50]	12 SSc	49 ± 4	Retrospective study (1998–2010)	HTPN support	5–270 months	↑ BMI: from 16.8 [12.3–21.3] kg/m^2^ to 18.3 [17.0–21.7] kg/m^2^ at 6 months and 19.7 [17.0-24.6] kg/m^2^ at 12 months No change in Karnofsky score (from 58.00 ± 3.27 to 39.00 ± 13.29 at 12 months)
[51]	25 SSc	55 [24–79]	Retrospective study (1990–1992)	PN support	3 months	Nutritional intervention duration: Mortality: 25% at 2-, 63% at 5-, and 77% at 10- years, but none directly from the PN Complication: catheter-related occlusion, sepsis, and thrombosis
[52]	5 SSc	62.2	Retrospective study (2008–2013)	PN support	12 months	↓Nutritional risk screening score (from 4.4 [4,5] to 1.4 [1,2] ↑BMI (from 19.1 [17.4–20.3] kg/m^2^ to 21.0 [18.3–23.4] kg/m^2^ ↑ Quality of life Complication: catheter-related infection (40%)

↑: increase; ↓: reduction; BMI: body mass index; EN: enteral nutrition therapy; GI: gastrointestinal involvement; HTPN: home total parenteral nutrition; low FODMAP: low-fermentable oligo-saccharide, disaccharide, monosaccharide, and polyol diet; N/A: not applicable; PN: parenteral nutrition therapy; SSc: systemic sclerosis patients.

### 3.6. Systemic Sclerosis and Predictors of Mortality

Seven papers [38,53,54,55,56,57,58] out of forty-nine analyzed attempted to identify predictors of mortality in patients with SSc (Table 5). In the seven articles, a total of 1357 SSc patients were analyzed. Since nutritional assessment is important in patients with SSc, malnutrition [38,53,57] and the malnutrition risk assessed with MUST were predictors of mortality [54]. Rosato et al. [38], Krause et al. [53], and Cruz-Domínguez et al. [57] highlighted malnutrition as a predictor of mortality, diagnosed with the GLIM criteria [38], the bioelectrical impedance analysis-derived phase angle (PhA) values [53], or the Chang index (calculated considering the variations in fat) [57], but not through the BMI [53]. Cruz-Domínguez and colleagues [57] also identified male sex and a severe Medsger’s score for general symptoms as among the risk factors for survival. In a single-center, prospective cohort study by Cereda et al. [54], the malnutrition risk of 160 SSc outpatients was assessed by MUST. A high nutritional risk was significantly associated with mortality, but only if it was evaluated according to MUST and not by anthropometric parameters. Prealbumin is also considered a mortality predictor [55]. In a multicenter, prospective study [55], participants were followed up for a median of 48 months and examined every 6 months. After the follow-up period, 34 patients had died. Age, male sex, lung, and GI involvement, as well as the presence of multiple visceral organ involvement, presence of co-morbidities, and low serum prealbumin, were significant predictors of mortality, with a higher hazard ratio for lung or multiple visceral organ involvement [55]. In Santosa et al.’s study [56], considering a population of 349 SSc patients from Singapore, after a mean follow-up of 2.1 years, 50% of deaths were attributed to SSc complication, and, in particular, to pulmonary arterial hypertension, ILD, and GI involvement. Smoking, SSc overlap, systolic arterial pressure, early renal involvement, vascular disease, and PN were independent predictors of mortality. Finally, in Rosato and colleagues’ study [58], PhA had predictive value in SSc patients, associated with an increased malnutrition risk, diagnosed both with MUST and GLIM, and decreased survival.

**Table 5:** Studies on predictors of mortality in systemic sclerosis.

Ref	Sample Size	Age (yrs)	Follow up	Predictor factors of Mortality	Hazard Ratio (95% CI)	*p*-Value
[36]	101 SSc	55 [47–66]	48 months	Malnutrition according GLIM criteria	4.38 (1.70–11.24)	0.002
[53]	124 SSc	54.0 ± 13.0	14.9 ± 6.7 months	Malnutrition	N/A	-
	295 HCs	48.2 ± 11.8				
[54]	160 SSc	62 [54–68]	46 months	Disease activity	6.3 (1.8–21.7)	0.004
				MUST	8.3 (2.1–32.1)	0.002
[55]	299 SSc	61 ± 11.8	48 [25–58] months	Low prealbumin	3.00 (1.52–5.97)	0.002
				Lung involvement	5.00 (2.45–10.34)	<0.001
				Multiple visceral organ involvement	3.94 (2.01–7.74)	<0.001
[56]	349 SSc	46.2 ± 4.1	2.1 years	Smoking	4.0 (1.5–10.6)	≤0.05
				SSc-overlap	6.0 (1.8–19.1)	≤0.05
				Baseline renal involvement	2.5 (2.2–11.7)	≤0.05
				PAP ≥ 40 mmHg	2.6 (1.1–6.5)	≤0.05
				Peripheral vasculopathy therapy	2.6 (1.1–6.5)	≤0.05
				Parenteral nutrition	8.8 (2.2–34.3)	≤0.05
[57]	220 SSc	51.16 ± 14.52	5.85 ± 4.10 years	Gener, male	5.84 (1.31–26.00)	0.020
				Severe malnutrition	3.77 (1.23–11.06)	0.021
				Severe general symptoms	5.12 (1.74–14-97)	0.003
[58]	104 SSc	55 [45–66]	48 months	Bioelectrical impedence analysis-derived phase angle	0.283 (0.083–0.965)	0.044

GI: gastrointestinal symptoms; GLIM: Global Leadership Initiative on Malnutrition criteria; HCs: healthy controls; MUST: Malnutrition Universal Screening Tool; N/A: not applicable; PAP: pulmonary artery systolic pressure; SSc: systemic sclerosis patients.

## 4. Discussion

This systematic review included articles focused on typical GI tract symptoms present in patients with SSc, with the aim of investigating the relationship between these, GM, and nutritional status and the nutritional interventions currently available to counteract the development of GI symptoms. Finally, we analyzed whether GI symptoms, GM alteration, and malnutrition could be predictors of mortality in these patients.

Evidence has increased in recent years, especially regarding the role of GM in the development of autoimmune diseases, including SSc. There is a bidirectional relationship between SSc and GI symptoms, as well as between SSc and GM. GI symptoms and GM alterations are manifestations of the disease, but in turn they influence its severity, damaging visceral organs or exacerbating the inflammatory state. Digestive tract fibrosis and dysbiosis alter nutrient absorption, which can lead to malnutrition. However, the articles that analyze these complex relationships between the GI tract, microbiome, and nutritional status are few and very heterogeneous. The same is true for the nutritional interventions to counteract digestive tract manifestations. Further studies are needed to investigate these complex interwinings and identify nutritional interventions able to reduce GI dysfunction and simultaneously counteract malnutrition, which has been shown to be a predictor of mortality.

SSc is a chronic disease of the connective tissue characterized by fibrotic degeneration of the skin and internal organs. Besides skin and lung involvement, the GI symptoms are frequently present (50–90% of the cases), often representing one of the earliest manifestations of the disease. GI symptoms involve the entire digestive tract from the oral cavity to the anus, with multiple manifestations, ranging from postprandial bloating, abdominal distension, abdominal pain, and diarrhea to anorectal dysfunction and severe malabsorption [2,3,5,60]. Due to microvascular damage, autonomic nerve dysfunction, muscular atrophy, fibrosis, and autoantibody production, 80% of the patients have impaired intestinal motility, a condition that predisposes them to bacterial proliferation. Bacteria deconjugate bile salts lead to malabsorption (50% of the cases) of lipids and fat-soluble vitamins. Moreover, bacteria digest sugars, producing gases and osmotically active products, thereby causing lactose malabsorption and diarrhea. Malabsorption in turn is usually related to the development of bacterial overgrowth and is among the causes of malnutrition. Malnutrition in turn contributes to disease severity and can be caused by mood disturbances, ILD, a poor functional status due to digital ulcers, reduced appetite, dysphagia, early satiety, microstomia, nausea, or other GI disturbances [36]. Malnutrition is highly prevalent in SSc, ranging from 8% to 55%, and is an independent risk factor for mortality [61]. This large variability in malnutrition prevalence is due to the different diagnostic tools used, such as BMI [24,25], MUST [62], bioelectrical impedance analysis parameters [58], the criteria issued by ESPEN [63], and new criteria proposed, such as GLIM [64], SNAQ [65], and 7-SGA [66]. Scientific evidence suggests that malnutrition is a potential modifiable risk factor for mortality. Therefore, a nutritional screening and assessment is of critical importance in the management of SSc patients.

From the studies analyzed, GM and GI symptoms are closely linked. Dysbiosis affects between 50% and 75.5% of patients with SSc. SSc patients present a different GM composition with respect to HCs, with a predominance of *Lactobacilli*, *Eubacterium*, and *Acinetobacter* and a decrease in *Roseburia*, *Clostridium*, and *Rinococcus* [12], as confirmed by several authors [6,15,65,66,67]. A recent review by Bellocchi et al. [68] provides an update on the GI microbiome in SSc. The authors discuss the role of GI microbiota in immune system development, its role in immunoregulatory functions and fibrosis onset, and its relationship with SSc-related GI dysfunction. Although food intake [16,69] and lifestyle, as well as the geographic area, can influence the GM, the differences found between SSc patients and HCs in GM composition are not totally attributed to food intake [13]. The composition of the microbiota has also been influenced by the worsening of GI symptoms over time, the duration of the disease, and the subtype of the disease [15], suggesting that alterations in the GM composition may precede the development of clinically manifest dysmotility in SSc. Individuals who develop ILD or SIBO associated to SSc have a different microbial composition than SSc patients without these complications [16]. Currently, only the association between the GI tract and ILD has been highlighted [70,71,72,73] and between the onset of SSc, the severity of ILD, and occupational exposure to crystalline silica and organic solvents [74]. It is not yet known how the microbiota can influence the onset of ILD. However, these results highlighted a link between distinct microbiome profiles and specific clinical manifestations of SSc. Future studies on the lung microbiome of SSc patients with varying severity of ILD may shed light on this issue and provide insight into the complex relationship between epithelial cells, immune cells, and fibroblast activation that culminates in tissue fibrosis [74]. Differences between SSc patients and HCs were also found in serum metabolites, also produced by the GM itself, with impacts on the host organism. For example, glycerophospholipids, the origin of which in SSc patients is still unknown, are bioactive metabolites that regulate several cellular pathways, including apoptosis and inflammation. For many molecules produced, especially by *Desulfovibrio*, it is not known whether they are the cause or the effect of the progression of the disease [13]. Butyrate is often produced at reduced levels in autoimmune diseases, including SSc [17]. It is essential for colonocyte energy, anti-inflammatory mechanisms, and maintenance of integrity of the gut barrier. Butyrate supplementation in patients with autoimmune diseases may have therapeutic benefits, but, despite encouraging results both in vitro and in animal models, clinical studies are limited and optimal strategies to modulate dysbiosis via its supplementation remain elusive [75].

The interactions between involvement and dysregulation in GM, metabolites produced, and development of GI, skin, and pulmonary complications in SSc are partly explained by the gut–skin axis [76], the gut–lung axis [77], and the duality between GM and the intestine itself. Although still partly undefined, several mechanisms of action have been hypothesized [78], also in light of the broader and less specific literature investigating the mechanisms through which the microbiota and host interact. The GM exerts numerous effects on the host metabolism, influencing several pathways [79]. Among these, the production of metabolites that can induce genetic and epigenetic modifications (alterations in DNA methylation and microRNA expression) or regulate neuroendocrine secretion, and the immune system, as well as intestinal membrane permeability, may be involved in the progression of SSc. Tryptophan (Trp) is metabolized in the intestine by the commensal bacterium *Lactobacillus reuteri*, resulting in the regulation of the immune system. In patients with SSc, the high concentration of *Lactobacillus* modifies Trp metabolism, with repercussions on the host immune defenses [79]. Additionally, patients with SSc have low concentrations of *Bacteroides* and *Anabaena* ssp., which have anti-inflammatory properties. This can suggest that the production of sphingolipids, which occurs by *Bacteroides* and is essential for maintaining intestinal homeostasis, is also deficient. *Faecalibacterium* and *Clostridium* are known to strengthen the epithelial barrier through the production of butyrate, also contributing to the regulation of mucosal inflammation. In those patients, *Faecalibacterium, Clostridium*, and butyrate production are lacking while pathogens, such as *Desulfovibrio*, which utilize butyrate to form harmful metabolites, thus contributing to its further deficiency, predominate [79]. Pathogenic mechanisms, such as microvascular damage, immune dysregulation, and multiorgan fibrosis, typical of SSc, are driven by alterations in microbiota. In the transition from homeostasis to disease, the Th17/Treg ratio is crucial and is significantly influenced by GM. Furthermore, GM also influences the immune system through pattern recognition receptors (PRRs), which can recognize pathogen ligands, activating pathways that lead to the release of inflammatory mediators (including tumor necrosis factor-α, interleukin (IL)-1β, IL-6, IL-10, leukotrienes). Thus, alterations in GM composition can lead to inflammation and fibrosis, potentially contributing to the progression of SSc. In turn, fibrotic processes can interfere with the production of beneficial short-chain fatty acid (SCFA). Furthermore, dysbiosis is often associated with a compromised integrity of the intestinal barrier, resulting in pro-inflammatory responses that can lead to tissue damage [79]. Pulmonary fibrosis, which is a complication in patients with SSc-ILD, may be promoted by metabolites produced by GM that can induce mesenchymal cell proliferation [79]. The same mechanisms may also underlie the bidirectional relationship between the gut and skin (the gut–skin axis). A compromised intestinal barrier allows microbes and metabolites to pass into the bloodstream, causing immune cell activation, reactive species and pro-inflammatory cytokine production, immune homeostasis disruption, and accumulation of extracellular matrix, contributing to skin damage or fibrosis [79]. Other mechanisms have also been hypothesized, more specifically related to the bidirectional relationship between GI symptoms and GM. The metabolites produced, such as SCFA (butyrate, acetate, and propionate), neuroactive compounds (i.e., serotonin precursors, GABA, and dopamine), bile acids, and succinate, can influence the intestinal motility and/or inhibit gastric emptying and secretion, through interaction with their intestinal receptors (FXR, TGR5, FFAR2, FFAR3) and subsequent release of glucagon-like peptide 1 and peptide YY. Therefore, the altered composition of GM in SSc patients can affect the production of these beneficial metabolites, also compromising the regulation of enteric nervous system activity, smooth muscle contractions [80], and the secretion of GI hormones [80], thus contributing to the GI dysfunction observed in SSc. In turn, impaired motility can cause stasis, creating an environment conducive to further microbial imbalance, with the proliferation of certain bacterial strains, which in turn perpetuate or worsen motility problems, creating a vicious cycle.

In light of these mechanisms, it is possible that gut dysbiosis and metabolic disruptions have an impact on disease progression. Given the observational nature of the data, we cannot however exclude some role of reverse causation, i.e., GM may be influenced by selected metabolites and biological changes rather than being their cause. Quantification of the role, if any, of reverse causation remains however undefined.

Hence, probiotics could represent an important therapeutic resource. They could therefore help, on the one hand, to correct GM imbalances and repair damage to the intestinal mucosa, restoring the intestinal barrier, and, on the other hand, they could improve skin diseases by reducing oxidative stress, inflammation, and extracellular matrix accumulation. However, the scientific evidence in favor of their use is still uncertain. Specifically in GI diseases, there are numerous studies, but the results on their application as a therapeutic means are often controversial or result from an inadequate study design [81,82]. Even in the specific case of SSc patients, the use of probiotics [19,20,21] to treat GI symptoms led to controversial results, with researchers recording no improvement [21] or a minimal effect on GI symptoms [19,20], particularly on reflux [19,20] and distention/swelling [19]. FMT may be a future approach in SSc [83]. The aim of FMT is to counteract dysbiosis and restore homeostasis between the host immune system and GM. While FMT has been successfully applied in *Clostridioides difficile* infections, uncertain results have been obtained in irritable bowel syndrome, inflammatory bowel disease, and SSc [83]. The negative results obtained by Fretheim and colleagues [23] could be attributed to the timing of FMT, type of FMT preparation, or instillation modality. However, this approach may also be limited due to the stability of the pre-existing host GM. More personalized interventions may be needed that allow, on the one hand, for replenishing the beneficial bacterial flora, and on the other hand, for eliminating the pathogenic flora, without damaging the rest of the GM. Moreover, the already complex relationship between the microbiome, GI symptoms, and clinical manifestations of SSc is complicated by the use of drugs, such as prokinetics, proton pump inhibitors, and antibiotics, which can alleviate some GI symptoms, but also can exacerbate others (diarrhea, malabsorption), negatively alter the microbiota (promoting SIBO and the growth of potentially pathogenic bacteria), and increase the risk of intestinal infections and systemic inflammation [84].

In addition to other influences, GI dysfunction can lead to malnutrition. The association between GI symptoms and malnutrition was confirmed in many of the studies considered [8,26,27,28,29,31,32,33,34,35,36,38,39,40,41,42], although there were exceptions [24,25,30,34,36,37]. The effects of malnutrition have been widely investigated [59,60,85,86] as it has a strong negative impact on quality of life, producing substantial co-morbidity and mortality. Among the screening tools used to assess the severity of malnutrition, MUST has proven to be a useful predictor of mortality for these patients [54]. According to the MUST screening tool, malnutrition is present in 18% of cases. Screening and management of malnutrition are very important, but only a few cases have evaluated the nutritional interventions in SSc patients, through the use of a targeted diet [17,43], nutritional counseling [44,46], or oral [45,46] or artificial nutrition support [47,48,49,50,51,52].

Regarding GI symptoms, the low-fermentable oligo-saccharide, disaccharide, monosaccharide, and polyol diet [43] led to a reduction in symptoms, whereas nutrition therapy (EN, PN) improved the self-reported nutritional status [46], but not GI symptoms (or health-related quality of life) [45]. Since oxidative stress is also one of the mechanisms that plays a role in cutaneous and visceral fibrogenesis, a nutritional intervention based on antioxidants, such as probucol, N-acetyl-l-cysteine, polyphenols, vitamins, and minerals, can be useful as an SSc support [87]. Specifically, calcium and 25OH-vitamin D supplements, but not zinc, selenium, or folic acid, could improve GI symptoms, since an association with GI tract involvement was highlighted [30,31]. A vitamin B12 deficiency is also possible in these patients, either due to alterations in the GI tract and the lack of production of intrinsic factors, which prevent its absorption, or due to intestinal dysbiosis, which alters its endogenous production. Monitoring vitamin B12 levels has therefore been proposed [28], but there is insufficient evidence to underline that supplementation can bring benefits. To the best of our knowledge, there are no studies on EN treatment, while the PN option can be used when ONS is insufficient or contraindicated in patients with a functional GI tract. Although the studies analyzed reported several complications after PN treatment, including catheter-related infection [47,49,51,52], calcification of the line [49,51], and reduction in autonomy due to the request for help in the daily management of PN [47,49,51,52], overall, PN improves the quality of life and reduces mortality. Given the paucity of studies, further studies regarding the modalities, benefits, and limitations of nutritional interventions in patients with SSc are needed. Currently, most of the useful information comes from diets used in the treatment of other immune-related inflammatory diseases, such as rheumatoid arthritis [88], psoriasis [89], and inflammatory bowel disease [90]. Nutritional interventions would be useful not only in trying to reduce GI symptoms but also in counteracting malnutrition, the most frequent predictor of mortality, together with others on several SSc-specific features [38,53,54,55,56,57,58].

The present systematic review has a few limitations. First, there is large heterogeneity in the included studies. Second, the majority of the included studies were retrospective and cross-sectional analyses. Third, several studies have relatively a small sample size. To limit the impact of these limitations on our final results as much as possible, the literature search was conducted carefully and by two independent readers.

## 5. Future Perspectives and Conclusions

Nutrition represents a fundamental tool that may be used to influence the microbiota composition, and the diet can be used as a therapeutic intervention. Further studies are needed to investigate the association between nutritional intervention and a clinical improvement in GI symptoms. Nutritional intervention based on the Mediterranean diet, whose beneficial effects on health have been extensively demonstrated elsewhere [91,92,93], or a vegetable-based diet [94] can lead to a reduction in GI symptoms. Perhaps they also act on the intestinal flora. The Mediterranean diet has a positive effect on GM [95]. The microbiome composition changes after only 24 h of dieting [69]. In particular, Bacteroidetes and Actinobacteria phyla were positively associated with fat, but negatively associated with fiber, while the *Firmicutes* and *Proteobacteria* genera had the opposite association. The clustering of GM taxa into enterotypes is also associated with the diet, but it takes ten days to change them. In particular, the *Bacteroides* enterotype was associated with animal protein, amino acids, and saturated fats, while the *Prevotella* enterotype was associated with a carbohydrate-based diet [16]. Equally, weight loss diets, characterized by a high protein intake and reduced content of total carbohydrates and fiber, followed for only 4 weeks, resulted in a significant decrease in fecal cancer-protective metabolites and increased levels of branched-chain fatty acids, phenylacetic acid, and N-nitroso compounds [69]. A long-term adherence to such diets can increase the risk of colonic disease [69]. The studies cited did not involve SSc subjects, but indicate that dietary habits, independently from demographic data, influence the GM composition. This could be the rationale for designing and conducting long-term longitudinal studies aimed at modifying the GM in order to ameliorate SSc-related GI symptoms.

In conclusion, the available scientific evidence outlines a deep and complex relationship between SSc progression, the GM composition, GI symptoms, and the patient nutritional status, which is not fully understood. Multidisciplinary management of patients with SSc is therefore recommended, and there is a need to implement studies focused on nutritional interventions, of any nature, from the counseling approach to a targeted diet and nutritional support. Further studies of greater quality and rigor, possibly RCTs, focused on GM, from its characterization to the possibility of intervening with it to improve GI symptoms, will also be necessary. This will help identify therapeutic approaches and strategies in order to improve GI tract management, and, consequently, the prognosis, healthcare costs, and quality of life of SSc patients.

## Data Availability

Data may be made available for collaborative studies upon reasonable request to the corresponding author.

## Supplementary Materials

The following supporting information can be downloaded at https://www.mdpi.com/article/10.3390/ijms26167685/s1.

## Abbreviations

The following abbreviations are used in this manuscript:

SSc	Systemic Sclerosis
HCs	Healthy Controls
GM	Gut Microbiota
GI	Gastrointestinal
SIBO	Intestinal Bacterial Overgrowth
VEDOSS	Very Early Diagnosis of Systemic Sclerosis
ILD	Interstitial Lung Disease
FMT	Fecal Microbiota Transplantation
UCLA GIT 2.0	University of California Los Angeles Scleroderma Clinical Trials Consortium Gastrointestinal Tract 2.0
ONS	Oral Nutrition Support
EN	Enteral Nutrition
PN	Parenteral Nutrition
MUST	Malnutrition Universal Screening Tool
ESPEN	European Society of Clinical Nutrition and Metabolism
GLIM	Global Leadership Initiative on Malnutrition
SNAQ	Short Nutritional Assessment Questionnaire
7-SGA	Subjective Global Assessment, 1–7 points
RCTs	Randomized Controlled Trials
SCFA	Short-Chain Fatty Acid
